# Differences in polyadenylation site choice between somatic and male germ cells

**DOI:** 10.1186/1471-2199-7-35

**Published:** 2006-10-12

**Authors:** K Wyatt McMahon, Benjamin A Hirsch, Clinton C MacDonald

**Affiliations:** 1 Department of Cell Biology and Biochemistry, Texas Tech University Health Sciences Center, 3601 4^th ^St, Lubbock, TX 79430-6540 USA

## Abstract

**Background:**

We have previously noted that there were differences in somatic and male germ cell polyadenylation site choices. First, male germ cells showed a lower incidence of the sequence AAUAAA (an important element for somatic polyadenylation site choice) near the polyadenylation site choice. Second, the polyadenylation sites chosen in male germ cells tended to be nearer the 5' end of the mRNA than those chosen in somatic cells. Finally, a number of mRNAs used a different polyadenylation site in male germ cells than in somatic cells. These differences suggested that male germ cell-specific polyadenylation sites may be poor substrates for polyadenylation in somatic cells. We therefore hypothesized that male germ cell-specific polyadenylation sites would be inefficiently used in somatic cells.

**Results:**

We tested whether pre-mRNA sequences surrounding male germ cell-specific polyadenylation sites (polyadenylation cassettes) could be used to direct polyadenylation efficiently in somatic cells. To do this, we developed a luciferase reporter system in which luciferase activity correlated with polyadenylation efficiency. We showed that in somatic cells, somatic polyadenylation cassettes were efficiently polyadenylated, while male germ cell-specific polyadenylation cassettes were not. We also developed a sensitive, 3' RACE-based assay to analyze polyadenylation site choice. Using this assay, we demonstrated that male germ cell-specific polyadenylation cassettes were not polyadenylated at the expected site in somatic cells, but rather at aberrant sites upstream of the sites used in male germ cells. Finally, mutation of the male germ cell-specific poly(A) signal to a somatic poly(A) signal resulted in more efficient polyadenylation in somatic cells.

**Conclusion:**

These data suggest that regulated polyadenylation site choice of male germ cell-specific polyadenylation sites requires one or more factors that are absent from somatic cells.

## Background

Polyadenylation occurs in three stages: polyadenylation site choice, cleavage of the pre-mRNA, and addition of the poly(A) tail to the newly formed 3' end [1,2]. The first step, polyadenylation site choice, can be defined as the functional assembly of the factors necessary for pre-mRNA cleavage onto the pre-mRNA to allow for efficient, accurate cleavage of the pre-mRNA (it has also been called the commitment step) [3-5]. Mutation of the pre-mRNA sequence elements involved in polyadenylation site choice [6-12], or mutation of the protein machinery involved in polyadenylation site choice [13-15], result in inefficient polyadenylation of the pre-mRNA. Consequently, inefficient polyadenylation prevents export of mRNA and decreases production of the protein encoded by that mRNA [1]. Therefore, polyadenylation site choice is an important first step in polyadenylation and is essential for optimal gene expression.

In mammalian somatic cells, the mechanism of polyadenylation site choice has been intensely studied [1,2]. A number of pre-mRNA sequences have been proposed to be important in choosing the site of polyadenylation [16-20]; however, two seem to play a prominent role in mammalian somatic cells. The first is the hexameric poly(A) signal (most often AAUAAA) found 15–30 bases upstream of the site of polyadenylation [6,21]. The other is the G/U-rich element found 20–40 bases downstream of the site of polyadenylation [11,22]. Together, these elements bind the multi-subunit cleavage and polyadenylation specificity factor (CPSF) [23] and cleavage stimulation factor (CstF) [11], respectively. Thus, the formation of this protein/RNA complex determines the polyadenylation site choice. The next step is cleavage of the pre-mRNA at the polyadenylation site (a process that requires additional factors, possibly including CPSF-73 [24]) followed by addition of the poly(A) tail [1,2].

However, a number of mRNAs use different polyadenylation sites in different tissues or developmental stages [25]. Changes in the composition of the protein polyadenylation machinery can invoke a change in polyadenylation site choice called alternative polyadenylation [13,26,27]. In addition, inclusion or exclusion of pre-mRNA sequences outside of the polyadenylation region (*i. e*., by changes in splicing pattern or the presence of a "stronger" polyadenylation site) may also affect polyadenylation site choice [25]. Therefore, changes in the protein/pre-mRNA complex involved in polyadenylation site choice can change where the poly(A) tail is added, and thereby affect gene expression.

We have noticed that the polyadenylation sites chosen in male germ cells are different from those chosen in somatic cells [28]. First, a number of mRNAs use a polyadenylation site at higher frequency than in other tissues [25,29-31]. Second, the incidence of the sequence AAUAAA near the 3' ends of male germ cell mRNAs is lower than in somatic mRNAs [28,32]. Third, the polyadenylation sites chosen in male germ cell mRNAs often result in shorter 3' untranslated regions than somatic mRNAs [32]. This suggests that there are significant differences in the polyadenylation sites chosen in somatic and male germ cells. There are two possible causes of these differences. Either male germ cell-enriched polyadenylation sites can be used in somatic cells but are not (because they are on pre-mRNAs not expressed in somatic cells, because other pre-mRNA elements prevent their use, or because the somatic polyadenylation sites out-compete them), or they are poor substrates for polyadenylation in somatic cells.

We hypothesized that male germ cell-specific polyadenylation sites are poor substrates for polyadenylation in somatic cells, and therefore would be used inefficiently in somatic cells. To test this, we developed a luciferase-based reporter assay to evaluate the polyadenylation efficiency of different sequences. We then used the assay to show that sequences surrounding male germ cell-specific polyadenylation sites (called polyadenylation cassettes) were inefficiently polyadenylated in somatic cells. Additionally, we developed a 3' RACE-based approach to analyze polyadenylation site positioning. Using this approach, we observed that mRNAs containing these male germ cell-specific polyadenylation sites were not polyadenylated at the site chosen in male germ cells. Rather, they showed aberrant polyadenylation upstream of the male germ cell-specific polyadenylation site. Finally, we showed that introduction of an AAUAAA (an element important to polyadenylation site choice in somatic cells) into a male germ cell-specific pre-mRNA allowed for more efficient polyadenylation of that site in somatic cells. These data suggested that male germ cell-specific polyadenylation sites were inefficiently chosen in somatic cells, and that polyadenylation site choice has different requirements in male germ cells than in somatic cells.

## Results

### Development of a luciferase reporter system to assay polyadenylation efficiency

To test whether male germ cell-specific polyadenylation sites were inefficiently polyadenylated in somatic cells, we developed a system to assay polyadenylation efficiency. We used PCR to isolate genomic sequences surrounding various polyadenylation sites used in vivo; we called these sequences polyadenylation cassettes (the primers used to make these cassettes are shown in Table 1). Each polyadenylation cassette was between 150 and 200 base pairs long, approximately centered on the site of polyadenylation. Each polyadenylation cassette was separately sub-cloned downstream of the coding region of Renilla luciferase (replacing the SV40 late cassette that is part of the reporter) to make a reporter plasmid. This reporter plasmid was then co-transfected into somatic cells (mouse embryonic fibroblasts) with a separate plasmid expressing firefly luciferase to control for transfection efficiency (see Materials and Methods). Since efficient polyadenylation is necessary for gene expression [6-8,33], relative luciferase activity (the quotient of Renilla and firefly luciferase activities) was used as a measure of polyadenylation efficiency for each polyadenylation cassette. Similar assay systems have been developed using the CAT reporter system [34,35].

To determine whether our assay measures polyadenylation efficiency, we tested three previously characterized polyadenylation cassettes. The first two, the SV40 late and the rabbit β-globin polyadenylation cassettes, are strong polyadenylation cassettes, and were therefore used as a reference for efficient polyadenylation. The third was a SV40 late mutant cassette, which was the result of mutating the poly(A) signal from AAUAAA to GAGAAA. Mutating the SV40 AAUAAA was previously shown to prevent binding of the polyadenylation machinery to the pre-mRNA, thus preventing polyadenylation site choice and resulting in inefficient polyadenylation [5,36,37].

**Figure 1:**
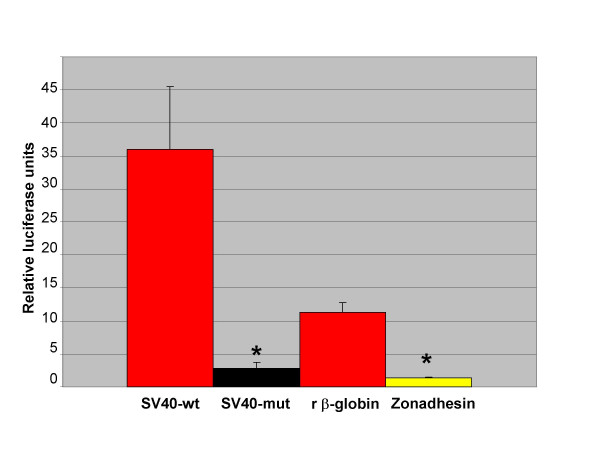
**The luciferase assay evaluates polyadenylation efficiency**. ATCC 3T3 cells (mouse embryonic fibroblasts) were transfected with *Renilla *luciferase reporter plasmids containing one of the indicated (bottom) polyadenylation cassettes as well as a firefly luciferase expressing vector to control for transfection efficiency. Two days later, extracts of these cells were assayed for luciferase activity. Indicated on the left is the relative luciferase activity (*Renilla *luciferase values divided by firefly luciferase values). Asterisks indicate significant difference from the SV40 wild type as determined by Student's T-test (p < 0.01).

We transfected mouse embryonic fibroblast cells (ATCC-3T3) with the above reporter plasmids and 48 hours later lysed the cells and assayed thy lysates for luciferase activity. Pilot experiments were performed to determine conditions for transfection that yielded a linear increase in Renilla luciferase activity (data not shown). As expected, lysates from cells transfected with reporter plasmids containing the wild type SV40 late or rabbit β-globin polyadenylation cassettes showed significantly higher levels of luciferase than those lysates from cells transfected with the mutant-containing reporter plasmid (Figure 1). Interestingly, lysates from cells transfected with reporter plasmids containing the SV40 late polyadenylation cassette showed 3.5 times higher levels of luciferase activity than those containing the rabbit β-globin cassette (Figure 1). This could be because the SV40 late polyadenylation cassette is viral, and therefore highly efficient in many tissues, whereas the rabbit β-globin cassette is being expressed in mouse fibroblasts rather than in rabbit reticulocytes (where it is normally expressed). These data demonstrate that the luciferase activity in lysates from these cells correlates with polyadenylation efficiency.

To test whether a male germ cell-specific polyadenylation cassette could be efficiently polyadenylated in somatic cells, another reporter plasmid was created with the polyadenylation cassette from the zonadhesin pre-mRNA. Since the zonadhesin mRNA is expressed only in male germ cells [38], it was used as a reference for male germ cell-specific polyadenylation. Similar to the results using the SV40 mutant reporter plasmid, the level of luciferase activity in lysates from cells transfected with the reporter plasmid containing the zonadhesin cassette was significantly lower than the level in lysates from cells transfected with the SV40-containing reporter plasmid (Figure 1). Therefore, the luciferase activity present in lysates of cells transfected with a reporter plasmid containing a male germ cell-specific polyadenylation cassette was similar to the level found in lysates from cells transfected with an inefficiently polyadenylated cassette.

### Genes with male germ cell-specific polyadenylation sites are expressed at lower levels than those with somatic polyadenylation sites from the same mRNA

**Figure 2:**
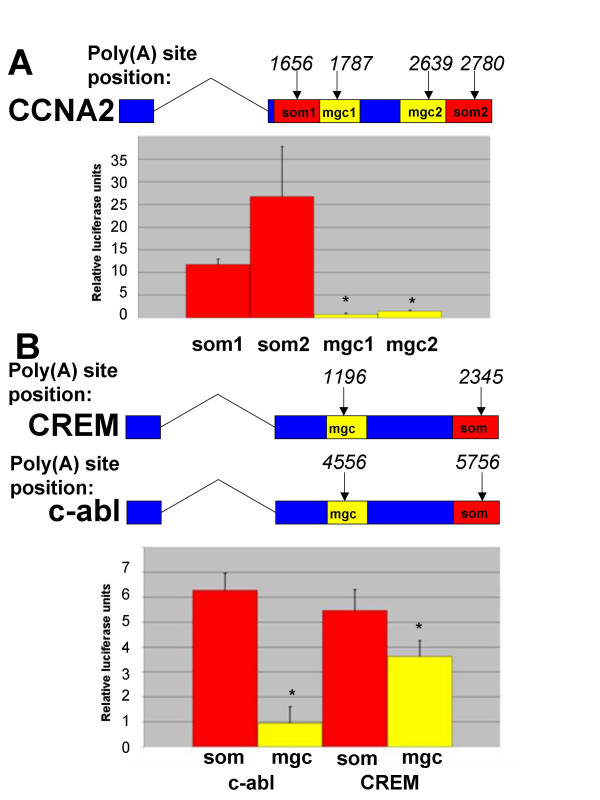
**Somatic polyadenylation cassettes are preferred tomale germ cell-specific cassettes**. A) At the top is a map indicating the positions of the various polyadenylation sites on the cyclin A2 mRNA. Above the diagram is the positions of the poly(A) sites used [27], and below is the name of the cassette. The diagram is approximately drawn to scale. Below, ATCC 3T3 cells (mouse embryonic fibroblasts) were transfected with plasmids containing somatic or male germ cell-specific cassettes indicated and extracts were assayed for activity as described in Figure 1. B) Same as A, but using CREM and c-*abl *cassettes. The poly(A) positions at the top are from [26, 28]. For both A and B, asterisks indicate significantly different from the respective somatic cassette as determined by Student's T-test (p < 0.01).

The SV40 wild type and rabbit β-globin polyadenylation cassettes are well-characterized [1,2]. In order to determine whether male germ cell-specific polyadenylation sites are truly inefficiently polyadenylated relative to somatic polyadenylation sites in somatic cells, we compared the polyadenylation efficiency of somatic and male germ cell-specific cassettes that both derive from the same gene. Since cyclin A2 potentially can use two different polyadenylation sites in somatic cells and two different polyadenylation sites in male germ cells [30], mouse embryonic fibroblasts were transfected with reporter plasmids containing either the somatic or the male germ cell-specific polyadenylation cassettes from the cyclin A2 mRNA. Luciferase activity from lysates of these transfected cells was then assayed. As shown in Figure 2A, lysates from cells transfected with the reporter plasmids containing the somatic-1 and somatic-2 polyadenylation cassettes from cyclin A2 showed much higher luciferase activity than did lysates from cells transfected with male germ cell-specific-1 and male germ cell-specific-2 polyadenylation cassette-containing plasmids. Therefore, for the cyclin A2 pre-mRNA, transfection of cells with reporter plasmids containing the somatic polyadenylation cassettes resulted in expression of higher levels of luciferase activity than those containing the male germ cell-specific polyadenylation cassettes.

These studies were extended to compare the polyadenylation efficiencies of the various CREM and c-*abl *polyadenylation cassettes, each of which uses one polyadenylation site in somatic cells and a different site in male germ cells [29,31]. We transfected fibroblasts with reporter plasmids containing polyadenylation cassettes from c-*abl *and CREM mRNAs. Again, lysates from cells transfected with reporter plasmids containing male germ cell-specific polyadenylation cassettes showed lower levels of luciferase activity than did those lysates with somatic cassettes in the reporter plasmids (Figure 2B). Thus, even when studied independently of each other, the somatic polyadenylation sites are associated with higher levels of luciferase activities in extracts from transfected cells.

### Male germ cell-specific polyadenylation sites are inefficiently used in somatic cells

We wanted to determine whether the low levels of luciferase activity we observed in extracts from cells transfected with reporter plasmids containing male germ cell-specific polyadenylation cassettes were because of problems with polyadenylation site choice. To do this, we transfected mouse embryonic fibroblasts with reporter plasmids used in earlier experiments, extracted RNA from the transfected cells, made cDNA, and subjected it to 3' RACE. The products were purified, cloned, and sequenced, and the sequences were aligned with the reporter plasmid with which these cells were transfected in order to determine the site of polyadenylation (see Methods and Materials).

**Figure 3:**
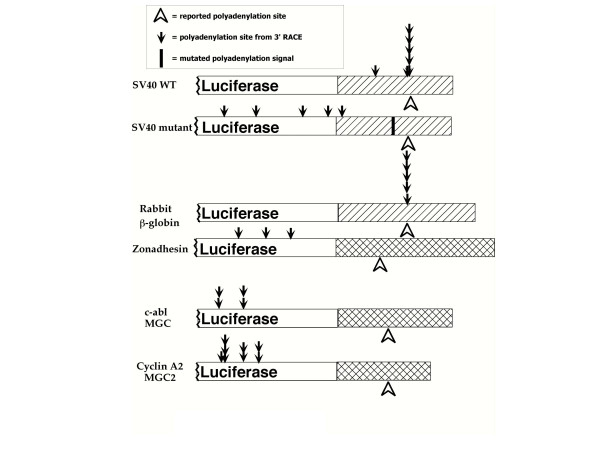
**Male germ cell-specific polyadenylation sites are not used in somatic cells**. ATCC 3T3 cells (mouse embryonic fibroblasts) were transfected with reporter plasmids containing the polyadenylation cassettes indicated on the left and two days later RNA was extracted from these cells, cDNA was made, and 3' RACE was performed as described in Methods and Materials. Shown are the results of the poly(A) site identification. The open arrow on the bottom indicates the reported site of polyadenylation on that polyadenylation cassette. The black arrows on top represent the sites of polyadenylation identified in transfected cells. The black bar at SV40-mutant shows a mutation in the poly(A) signal (AAUAAA to GAGAAA) of the SV40 polyadenylation cassette.

Figure 3 summarizes the results of these experiments. Of the seven cDNAs we cloned from cells transfected with reporter plasmids containing the SV40 late polyadenylation cassette, five were polyadenylated at the reported site, and the other two were within 100 bases of the reported site. Similarly, five of the five cDNA cloned from cells transfected with reported plasmids containing the rabbit β-globin cassette were polyadenylated at the reported site of polyadenylation.

In contrast, all five cDNAs cloned from cells transfected with the SV40 late mutant polyadenylation cassette-containing reporter plasmid showed polyadenylation at aberrant, upstream positions, and never at the reported site of polyadenylation (Figure 3). This upstream polyadenylation is different from the read through transcripts most investigators have observed because of inefficient polyadenylation [39-41]. In addition, the cDNAs cloned from cells transfected with reporter plasmids containing male germ cell-specific polyadenylation cassettes all showed aberrant upstream polyadenylation, similar to that seen with the SV40 mutant. Interestingly, most of the aberrant polyadenylation sites were found in the luciferase coding region. Therefore, all of the polyadenylation cassettes associated with low levels of luciferase give rise to aberrantly polyadenylated mRNAs when transfected into somatic cells.

### Sequences in cassettes affect polyadenylation efficiency

*Cis*-acting sequences around the site of polyadenylation have been shown to affect polyadenylation efficiency profoundly [3-9]. We hypothesized that the reason male germ cell-specific polyadenylation sites were not chosen in somatic cells was because they lacked the necessary *cis*-acting sequences that somatic polyadenylation site choice requires. If this were true, altering the sequences on a male germ cell-specific polyadenylation cassette would increase the ability of a male germ cell-specific polyadenylation site to be chosen in somatic cells.

**Figure 4:**
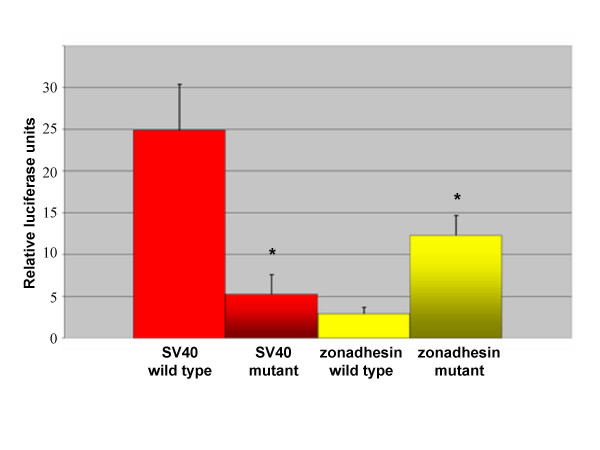
**The poly(A) signal is essential for polyadenylation of male germ cell-specific polyadenylation sites**. ATCC 3T3 cells (mouse embryonic fibroblasts) were transfected with reporter plasmids containing the indicated polyadenylation cassettes. Bars represent the average relative luciferase activity over three different replications. Asterisks indicate significant difference from the wild type version of each cassette (p < 0.01).

To test this hypothesis, we created four polyadenylation cassettes. Three of them (SV40 late wild type, SV40 late mutant, and zonadhesin) were used in earlier studies. We also created a zonadhesin mutant, which converted a putative male germ cell-specific poly(A) signal GAGAAA to an AAUAAA. Cells were transfected with reporter plasmids containing each of these four polyadenylation cassettes and subsequently lysates were assayed for luciferase activity. As before, cells transfected with the SV40 late-containing reporter plasmid showed high levels of relative luciferase activity, and lysates from cells transfected with SV40 late mutant and zonadhesin-containing reporter plasmids showed significantly lower levels of relative activity (Figure 4). However, when cells were transfected with the reporter plasmids containing a zonadhesin mutant polyadenylation cassette, significantly higher levels of luciferase activity were observed when compared to the zonadhesin-containing reporter plasmid. Therefore, altering the poly(A) signal in a male germ cell-specific polyadenylation cassette can increase the luciferase activity in extracts from cells transfected with this plasmid.

## Discussion and Conclusion

Our observations [28,42,43], as well as studies done by others [32,44], suggested that there were differences in how somatic and male germ cells choose a polyadenylation site. However, we had never tested directly whether somatic cells were capable of efficiently choosing a polyadenylation site from a male germ cell-specific mRNA. We therefore devised a luciferase reporter assay that measures polyadenylation efficiency. Using this assay, we showed that extracts from cells transfected with reporter plasmids containing male germ cell-specific polyadenylation cassettes showed significantly lower levels of luciferase activity than extracts from cells transfected with somatic cassette-containing plasmids. Extracts of cells transfected with reporter plasmids containing somatic polyadenylation cassettes also expressed higher levels of luciferase activity than those with male germ cell-specific polyadenylation cassettes from the same mRNA. Analysis of 3' RACE products showed that mRNAs containing somatic polyadenylation cassettes were polyadenylated almost exclusively at the reported sites. Mutation of the AAUAAA polyadenylation signal to a GAGAAA resulted in no observed polyadenylation at the reported sites. Rather, analysis of the 3' ends of these mRNAs showed that any mRNAs that were being polyadenylated were being polyadenylated at aberrant sites. Finally, introduction of an AAUAAA into a male germ cell-specific polyadenylation cassette resulted in significantly higher levels of luciferase in extracts transfected with this plasmid. These data suggest that male germ cell-specific polyadenylation sites are inefficiently used in somatic cells.

Each of the male germ cell-specific polyadenylation sites shown here are efficiently used in male germ cells [29-31]. Additionally, we have shown that the somatic polyadenylation sites for these pre-mRNAs are used in somatic cells (data not shown). Why, then, are male germ cell-specific polyadenylation sites inefficiently used in somatic cells? As discussed above, polyadenylation site choice is the product of the sequences present on the pre-mRNA and the polyadenylation machinery present in the cell. There is a lower incidence of the sequence AAUAAA near the 3' ends of mRNAs in male germ cells relative to the incidence in other tissues [28]. Other studies have suggested that other elements essential for polyadenylation in somatic cells may likewise be absent from male germ cell mRNAs [32]. The fact that male germ cell-specific polyadenylation cassettes often lack the sequences necessary for somatic polyadenylation could be the reason for male germ cell-specific polyadenylation sites not being chosen in somatic cells. Additionally, it is possible that elements in male germ cell-specific polyadenylation cassettes prevent their polyadenylation in somatic cells. However, this study has shown that male germ cell-specific polyadenylation sites are used rarely, if at all in typical somatic cells (Figure 3). Introduction of a somatic polyadenylation signal (AAUAAA) can greatly increase its ability to be chosen in these cells (Figure 4), but there are likely other elements involved as well. This suggests that the polyadenylation machinery present in somatic cells is less compatible with the polyadenylation sequences present in male germ cell-specific mRNAs than with somatically expressed mRNAs.

Additionally, male germ cells express proteins that are present either exclusively or predominantly in this tissue that are homologous to known polyadenylation proteins [42,43,45-47]. Two of these proteins, a CstF-50 homolog called WDC146 [45] and a CstF-64 homolog called τCstF-64 [42,43], are similar to proteins that have been associated with changing polyadenylation site choice [13,27,48]. The presence of these proteins in male germ cells may alter the polyadenylation machinery's preference for polyadenylation site choice, and their absence may explain why male germ cell-specific polyadenylation sites are inefficiently chosen in somatic cells.

We originally hypothesized that τCstF-64 would affect polyadenylation site choice for the mRNAs studied here [43]. However, we have recently shown that the CREM, c-*abl*, and CCNA2 mRNAs are expressed at normal levels and are polyadenylated at the same position in the testis of mice lacking τCstF-64 as in the wild type testis (KWM, B. Dass, T. Denison, K. Hockert, CCM, in preparation). This strongly suggests that τCstF-64 is not involved in changing the polyadenylation site choice for these transcripts. However, other proteins expressed in male germ cells – proteins that are also absent from fibroblasts – could be responsible for the change in polyadenylation site choice for these male germ cell-specific mRNAs.

One surprising finding was of the existence of aberrantly polyadenylated mRNAs with poly(A) site choice occurring upstream of the reported site of polyadenylation (Figure 3). Previous studies showed that if a pre-mRNA was inefficiently polyadenylated, then RNA polymerase II read through the site of polyadenylation, producing longer transcripts [39-41]. In contrast, we observed mRNAs that were polyadenylated at disparate places, all upstream of the reported site and several in the luciferase coding region. We used a 3' RACE-based approach to look for changes in polyadenylation; this is opposed to the RNase protection assays and nuclear run-on assays previous investigators have used [41,49,50]. Because we saw this aberrant polyadenylation associated with the SV40 late mutant polyadenylation cassette, we believe that aberrant polyadenylation is the result of inefficient polyadenylation. It is also possible that these aberrant polyadenylation sites often occur naturally, but are only detectable in the absence of efficient polyadenylation. However, we propose that the use of a more sensitive PCR-based method has allowed us to observe previously undescribed upstream aberrant polyadenylation.

Finally, our data suggest the possibility that male germ cells express one or more different factors and have different sequence requirements that together alter how the polyadenylation site is chosen. Other tissues may similarly have different mechanisms of polyadenylation site choice. The brain seems especially prone to contain mRNAs that are alternatively polyadenylated [44], and our lab has identified a brain-specific form of CstF-64 (G. Shankarling, CCM, in preparation), which may alter polyadenylation site choice in this tissue. The existence of such alternative methods of polyadenylation site choice could be used to greatly increase the protein diversity within a cell.

## Methods

### Cell culture

Mouse ATCC-3T3 cells were grown at 37°C in growth media (Gibco Dulbecco's Minimal Eagle Media in the presence of Gibco 10% newborn calf serum and penicillin/streptomycin).

### Reporter plasmids

Each of the cassettes was created by using PCR to amplify polyadenylation cassettes from a bacterial artificial chromosome (BAC) obtained from the Children's Hospital Oakland Research Institute (CHORI). The primers and BAC templates used for the PCR reactions are shown in Table 1. Each of the 5' primers contain also contains an XbaI (TCTAGA) site at the 5' end and each of the 3' primers contain a BamHI site (GGATCC) at the 5' end. These products were cloned into pCRII using the TOPO cloning kit (Invitrogen, Carlsbad, CA) according to manufacturer's instructions. pGL3-promoter (Promega, Madison, WI) was used as the transfection control. Mutations were done using the QuickChange site directed mutagenesis kit from Promega, per manufacturer's instructions.

The various reporter plasmids were created by subcloning (using standard subcloning techniques) each of the polyadenylation cassettes separately into the pRL-SV40 reporter plasmid using the XbaI and BamHI sites that surround the SV40 polyadenylation cassette that is a part of the plasmid. In doing so, the SV40 polyadenylation cassette was removed from each of the newly-made reporter plasmids.

### Luciferase assays and transfections

For luciferase assays, cells were plated on 24-well dishes at 1.5 × 10^4 ^cells/well 16–24 hours before transfection. 12 ng of pRL-SV40 plasmid (containing polyadenylation cassette of interest) 4 ng of pGL3-promoter plasmid, and 500 ng of pBluescript (used as a carrier plasmid) were mixed together and transfection with Lipofectamine™ was carried out per manufacturer's instructions (Invitrogen). The same transfection was done in six different wells for each experiment, making 6 replicates.

After 48 hours, cells were washed three times in PBS and lysed in 100 μL of lysis buffer from the Dual Luciferase Reporter Assay System (Promega) for 15 minutes shaking at room temperature. The lysates were then removed from the wells with a micropipette and transferred to a clean microcentrifuge tube on ice. Lysates were either used immediately or stored at -80°C before being thawed on ice. Luciferase assays were done according to manufacturer's instructions and luciferase activity was measured on a TD-20/20 (Turner Designs, Sunnyvale, CA). All values were recorded on a Microsoft Excel spreadsheet and calculations were done within this document. The numbers reported are in relative luciferase units, which is the ratio of *Renilla *luciferase activity to firefly luciferase activity. The value for each experiment was the average relative luciferase activity of the six different replicates. The numbers reported are the averages of three separate experiments (making a total of 18 different replicates), with standard deviations illustrated using error bars.

### 3' RACE

For 3' RACE, RNA was extracted from transfected cells using Trizol (Invitrogen) and treated with DNAse, then purified by phenol/chloroform and ethanol precipitation. 1 μg of DNA-free RNA was converted to cDNA with the SMART RACE kit (BD Biosciences), using 1/10 the amount of oligo(dT) the manufacturer recommends. 1 μL of a 1:10 dilution of cDNA was then used as a template for the first round of PCR which used the 5' primer (5'-GAACCATTCAAAGAGAAG-3') and the Universal Primer A mix. The resulting reaction was diluted 1:10 in ddH_2_O and 1 μL was used in a second round of PCR that included the 3' primer (5'-GTGAAGTTCGTCGTCCAAC-3') and Nested Universal Primer A.

Following both rounds of amplification, the products were separated by agarose gel electrophoresis, purified, and cloned into pCRII using the TOPO cloning kit (Invitrogen) according to the manufacturer's instructions. Multiple (between 5–10) clones were sequenced and aligned to the pRL-SV40 reporter plasmid sequence to determine the position of the poly(A) tail. A poly(A) tail was said to be legitimate (not due to false priming of the oligo(dT) primer) if it met the standards of the polya_db [49].

## Authors' contributions

KWM created the SV40 mutant, zonadhesin, rabbit β-globin, cyclin A2, and zonadhesin mutant polyadenylation cassette-containing reporter plasmids, determined optimal conditions for all of the assay systems, did the experiments, analyzed the data, and wrote the paper.

BAH determined the optimal conditions for creating the polyadenylation cassettes and made the CREM and c-*abl *polyadenylation reporter plasmids.

CCM developed the ideas for all of the assay systems and experiments, provided extensive guidance, and was vital to the production of the final paper.

All authors read and approved the final manuscript.

## Figures and Tables

**Table 1:** Primers used in the creation of polyadenylation cassettes.

**Cassette/Template**	**Primer sequence**
CCNA2-SOM1/336M6	5'-GT**TCTAGA**CTGCTTTGTTTGCCCATTTTTCTC
	3'-CTT*GGATCC*CTCCCCACTGTACAATAAAACAA
CCNA2-SOM2/336M6	5'-AATT**TCTAGA**GGTTAACATTGGTGCTGCCAAC
	3'-GTTT*GGATCC*CCCTGAGCAATGAAAGTGGAC
CCNA2-GC1/336M6	5'-G**TCTAGA**GTAGGAAAAGATTTTATAAAAC
	3'-CAT*GGATCC*CTGTAACATCTGAGAGCCGATC
CCNA2-GC2/336M6	5'-C**TCTAGA**GAATCTTACCCTGTTCTAAGTAAG
	3'-C*GGATCC*CATGGGCAGTCCTGGTAGCAAG
CREM-somatic/304K8	5'-**TCTAGA**GTCACGTAGGTCTAGACTGGC
	3'-*GGATCC*CTGAGTTCGGATCCCAACACC
CREM-MGC/304K8	5'-**TCTAGA**AACAAGAAGCTTCTAGAGGAGC
	3'-***GGATCC***CCTTAGAGGTAGGATCCAATTAAATAC
c-abl-somatic/156H9	5'-**TCTAGA**CCAGTACATTTTGTTTCTAGATATGACTG
	3'-*GGATCC*CTGCTGTGAGGATCCCAAGAG
c-abl-mgc/156H9	5'-**TCTAGA**TTCCCTGTCCTTCTAGAAC
	3'-*GGATCC*GATGGACCTTCTAGACACCAAG

Shown at left are the names of the polyadenylation cassettes with the BAC template used as a template to create each cassette, and at right the sequences of the primers used to make the respective polyadenylation cassette. The XbaI and BamHI restriction enzyme sites used for cloning are shown in bold and italics, respectively.
